# Postoperative pain after pulpotomy versus pulpectomy of primary molars with symptomatic irreversible pulpitis: an equivalent randomized clinical trial

**DOI:** 10.1186/s12903-026-08329-z

**Published:** 2026-04-25

**Authors:** Dania Ibrahem Sermani, Mahmoud Ahmed Abdelmotelb, Ahmad Abdel Hamid Elheeny

**Affiliations:** 1 Faculty of Dentistry, Minia University, Minya, 61519 Province Egypt; 2 Faculty of Dentistry, Sphinx University, Minya, Egypt

**Keywords:** Postoperative pain, Pulpectomy, Pulpitis, Pulpotomy

## Abstract

**Objectives:**

The study aimed to compare the incidence of postoperative pain after pulpotomy versus pulpectomy of primary molars with symptomatic irreversible pulpitis (SIP).

**Materials and methods:**

An equivalent parallel, two-tailed randomized controlled trial was conducted involving 92 children aged 5 to 7 years presenting with carious mandibular second primary molars exhibiting signs of SIP. Participants were randomly assigned to two equal groups of 46 children each, with the affected mandibular second primary molars treated using either pulpotomy as the intervention or pulpectomy as the control treatment. The postoperative severity of pain was determined at 6, 12, 24, 48, 72 h and after one-week following treatment using a modified Wong-Baker FACES (WBF) pain scale. Considering an equivalence margin of 10% between the two endodontic techniques, the absolute risk difference (ARD) was calculated at a 90% confidence interval. The level of significance was set to < 5%.

**Results:**

After six hours, 65.2% of pulpotomy and 73.9% of pulpectomy patients reported no pain. At 24 and 48 h, pain absence was comparable between groups (73.9% vs. 78.3%). However, by one week, 8.7% of pulpotomy patients still experienced severe pain versus 2.2% in pulpectomy. The incidence of postoperative pain severity after either pulpotomy or pulpectomy was equivalent with no significant difference over the follow-up periods.

**Conclusions:**

Pulpotomy could be an alternative approach to treat primary molars with SIP when hemostasis can be achieved.

**Clinical relevance:**

Postoperative pain intensity is a critical factor in assessing the success of endodontic treatment, especially in children. Pulpotomy can be a suitable and less invasive endodontic treatment for primary teeth with SIP.

**Supplementary Information:**

The online version contains supplementary material available at 10.1186/s12903-026-08329-z.

## Introduction

Pain experience during or following dental procedures is one of the determinant factors that shape the child's pain experience, behavior toward dentistry, and dental fear in the later age stages. Moreover, pain experienced in childhood not only has a negative impact on the child but also has a distressing influence on the child's parents or caregivers [1].

Vital pulp therapy (VPT) in terms of pulpotomy or pulpectomy in pediatric patients is a common procedure in pediatric offices and is associated with a high incidence of postoperative pain [2]. Pulpectomy is the treatment choice for primary molars with irreversible pulpitis. However, some disadvantages can be encountered with pulpectomy, including sacrificing more tooth structure, technique difficulties, and a longer time, which is a critical factor affecting the child's behavior [3–5]. Pulpectomy in primary molars is a complicated procedure due to the complex nature of the root canal anatomical system, which makes total root canal debridement impossible. Additionally, it is inevitable that the extrusion of foreign material from the apical foramen will trigger a periapical inflammatory process and subsequent post-endodontic pain after the chemo-mechanical manipulation [6]. In contrast, pulpotomy is a less invasive approach that relieves pain before starting the definitive pulpectomy and allows for the preservation of the viability of remaining pulp tissues after capping with a biocompatible material [7].

The diagnosis of irreversible pulpitis in children is a challenging task. Clinical diagnosis of primary teeth with symptomatic irreversible pulpitis (SIP) relies primarily on the patient’s symptoms especially with lack of reliability of thermal pulp testing in primary teeth. Accordingly, the clinical diagnosis of SIP relies on the duration of pain, particularly its persistence following the removal of thermal stimuli and/or its spontaneous occurrence in the absence of an identifiable trigger [8, 9]. Conventionally, based on these subjective indicators of SIP, pulpectomy is the treatment of choice, especially in primary teeth. Pulpectomy decision is related to the firm conviction of the poor healing capability of the pulp of primary teeth due to the extension of inflammation to the radicular pulp tissues and subsequent widespread of the inflammatory process in response to carious exposure [10].

However, the recent trend towards streamlining complicated endodontic procedures (i.e. minimally invasive endodontics) by levering the potential of pulp tissue healing and even repair. The better understanding of pulp pathophysiology in conjunction of the introduction of new bioactive calcium silicate cements, permit considering less invasive endodontic pulp therapy approaches such as pulpotomy to treat teeth exhibited signs and symptoms of irreversible pulpitis. Pulpotomy provides intraradicular favorable healing conditions that prompts the healing capacity of pulp tissues [11]. Meanwhile, most of these histological and clinical trials are concerned with permanent teeth and little is known about the clinical success of pulpotomy instead of pulpectomy in treatment of primary teeth with irreversible pulpitis.

Immunological and histological findings in terms of neurovascular changes elucidate that the response of pulp tissues of primary teeth is mimicking the response of pulp tissues of permanent teeth [12, 13]. Accordingly, utilizing this similarity in pulp response between primary and permanent teeth to caries may pave the way for employing pulpotomy instead of pulpectomy in the treatment of primary tooth pulp. Employing pulpotomy instead of pulpectomy in primary teeth might be beneficial, potentially preserving more tooth structure and promoting better outcomes.

Until now, there has been little data available regarding the postoperative pain incidence of pulpotomy in treatment of primary molars. Therefore, the current prospective clinical trial has been conducted to assess the incidence of postoperative pain intensities following pulpotomy of primary teeth with symptomatic irreversible pulpitis compared to traditional treatment with pulpectomy. The study aimed to compare the incidence of postoperative pain after pulpotomy versus pulpectomy of primary molars with SIP. The hypothesis of the current equivalent randomized clinical trial suggested the incidence of postoperative pain after pulpotomy versus pulpectomy of primary molars with SIP was equivalent.

## Materials and method

### Ethical aspect

Procedures involving human participants were performed and reviewed according to the regulations of the Ethics Committee of the local institution (Reference no. 1029 on the 24th of February 2025). The trial was registered on ClinicalTRial.gov (ID NCT06903013 on 24th of March 2025). All procedures were performed in accordance with the 1964 Helsinki Declaration and its later amendments or comparable ethical standards.

### Study setting and design

**Fig. 1:**
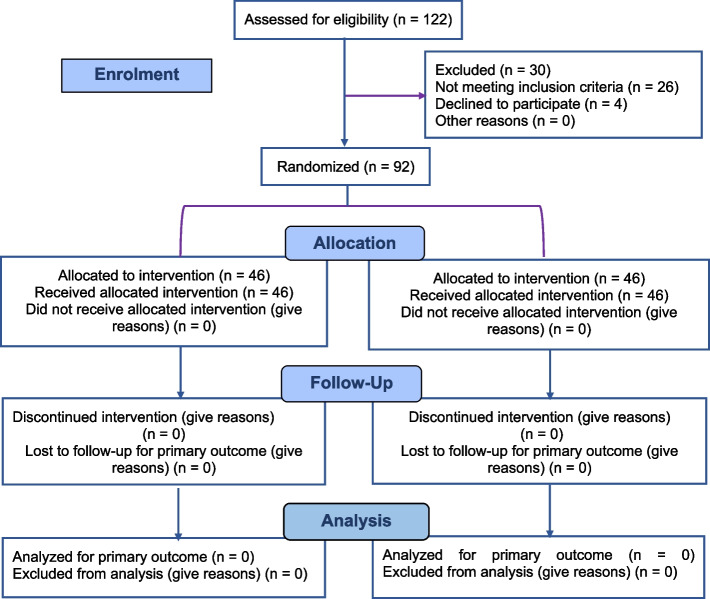
CONSORT flow diagram of the study design

The trial was held in the outpatient clinic of the Pediatric Dentistry Department, Hospital of the Faculty of Dentistry, Minia University. The trial was designed as an equivalent two-tailed randomized control trial with two parallel arms. The study adheres to the Consolidated Standards of Reporting Trails (CONSORT) guidelines for randomized clinical trials [14] (Fig. 1).

### Sample size, randomization and allocation

According to the findings of a pilot study that included 20 children (10 children per group), the difference in postoperative pain proportions between the two groups (∆) was 0.15. At an alpha level of significance of 0.05 and a power of 0.08, a total of 92 children were required after considering a non-response bias of 25%.

The participants were randomly allocated into two equal groups (46 children per group), using permutated block randomization. An independent qualified nurse had prepared a list using an online software “https://www.sealedenvelope.com/simple-randomiser/v1/listsGenerating”. Another independent nurse prepared ninety-two identical nontransparent envelopes that contained four-time folded sheets (8.27 × 11.69 inches). Each sheet contained a code representing the treatment technique and wrapped within aluminum foil sheets. Considering an allocation ration of 1:1 and according to the VPT technique, the envelopes were shuffled then placed in two identical labelled containers. At the time of intervention, four sealed allocation envelopes (two from each treatment container) were withdrawn and shuffled. A caregiver, blinded to their contents, randomly selected one envelope, and the treatment indicated inside was assigned to the participant. Children were equally allocated into two groups: pulpotomy “intervention” group and pulpectomy “control” group (46 participants per group).

### Inclusion and exclusion aspects

#### Inclusion criteria

Included children 5 to 7 years, weighed > 20 kg [8, 15] with free medical history and apparently healthy (class 1 or 2 according to the ASA classification). Only cooperative children (rate 3 or rate 4 based on the Frankl behavior rating scale) with no previous dental experience were enrolled in the trial. Regarding the teeth included, only mandibular second primary molars with deep carious lesions and suffered from the signs and symptoms of irreversible pulpitis were included.

The diagnosis of SIP was confirmed clinically via (1) confirming a positive history of spontaneous lingered pain that has been precipitated by thermal provocation [16] and (2) teeth pulps were early responded to the thermal pulp test (TPT). These responses were recorded after obtaining the baseline records of TPT of the contralateral tooth. Teeth with negative radiographic findings (i.e. no apical or furcal evidence of radiolucency) and ≥ two-thirds of the root length were present were included.

Additionally, before starting the procedures, only children who experienced severe pain intensities based on their records on the pain intensity scales were included to ensure the condition of SIP.

#### Exclusion criteria

Children with severe intellectual, behavioral, emotional, or concerns were excluded. Children with analgesic intake in the preceding 12 h were excluded. Moreover, non-restorable crowns were excluded. Clinically, a 5-min cut-off was used to identify cases with uncontrolled pulpal bleeding that are unlikely to respond to vital pulpotomy and therefore require pulpectomy or extraction. Additionally, teeth with abnormal tooth mobility, fistulous tract, or mucobuccal fold swelling and/or radiographic indicators of furcal/periapical pathology or internal root resorption were excluded.

### Clinical procedures

All clinical steps were performed by a single operator (postgraduate pediatric dentist student with an experience of 5 years). Pulp therapy approaches (pulpotomy and pulpectomy) were performed in a single appointment. Before starting the clinical procedures, nonpharmacological behavior management techniques, including tell-show-do and distraction strategies, were utilized to facilitate patient cooperation.

#### Pulp sensibility testing

The tooth in the contralateral side was isolated with a cotton roll that has been placed in the mucobuccal fold, then tested to provide a baseline to facilitate the comparison. Thermal testing using a Green Endo-Ice refrigerant (Coltene/Whalkedent Inc., Cuyahoga Falls, Ohio, USA) that was sprayed over a small cotton pellet and placed over the middle third of the buccal tooth surface for five seconds [8, 17].

#### Local anesthesia and rubber dam placement

Topical anesthetic was applied prior to injection. An inferior alveolar nerve block (IANB) was administered to anesthetize the carious primary mandibular second molars, using a 27-gauge long needle (C-K JECT®, Gyeonggi-do, Korea; 0.40 mm) with an aspirating syringe. The dosage of 2% lignocaine hydrochloride with 1:100,000 epinephrine (Lignospan® Standard, 1.7 mL, SEPTODONT Ltd.) was calculated based on the child’s body weight, ensuring it did not exceed the maximum recommended dose of 4.4 mg/kg [18]. Standard IANB injection technique was considered. After determining the anatomical landmarks, the needle was inserted, and anesthetic solution was deposited slowly over 60 s. To facilitate the rubber dam clamp, the lingual soft tissues were anesthetized by depositing a few drops while retracting the needle and the long buccal nerve was anesthetized after confirming optimal signs and symptoms of local anesthetic drug.

#### Caries removal and access cavity preparation

Following caries excavation, a straight-line access cavity was established using a no. 245 bur (Dentsply Maillefer, Tulsa, OK, USA) in conjunction with an Endo-Z bur to ensure proper canal entry.

#### Pulpotomy (intervention) group

The coronal pulp tissue was carefully amputated up to the level of the root canal orifices. The access cavity was checked for any caries residuals, then disinfected with 1.5% sodium hypochlorite (NaOCl). Hemostasis was attained using a cotton pellet soaked in NaOCl for 30 s. A sterile cotton pellet soaked with saline was inserted into the pulp chamber for 2 min against the root canal orifices. The access cavity was finally checked for hemostasis. If bleeding continued, the irrigation with 1.5% NaOCl followed by cotton soaked with saline was applied for another 1 min with a hemostasis time of 5 min as cut-off time [19]. After achieving hemostasis and according to the manufacturer’s instructions, a fast set powder and liquid MTA-based cement (RetroMTA, BioMTA, Seoul, Korea) was mixed and introduced over the pulp stump. After 12 min (the recommended setting time based on the manufacturer’s guidelines).

#### Pulpectomy (control) group

A manual stainless-steel K-file size #10 (MANI Inc.) was utilized to establish and confirm a patent glide path to the full working length (WL) of the root canals. Subsequently, root canal instrumentation was performed using the crown-down technique. A single-file 25/0.06 instrument was operated at a rotational speed of 400 rpm with a torque setting of 1 N·cm, utilizing a 6:1 reduction contra-angle handpiece powered by an X Smart Plus endomotor (Dentsply Maillefer). Mechanical preparation was carried out according to the previously established working length (WL) in three sequential steps: to two-thirds of the WL, to 3 mm short of the WL, and finally to the full WL. A gentle pecking motion was employed throughout instrumentation, with no apical pressure applied, to ensure controlled advancement to the WL [3]. All files were lubricated with 17% EDTA gel (Dolo®, Prevest DenPro, India). Irrigation was performed using 1% sodium hypochlorite between each instrument, delivered with 30-gauge side-vented irrigation needles (Prime Dental Products, India). Each root canal was flushed with 5 mL of normal saline, dried using sterile paper points, and subsequently obturated with Metapex (Meta Biomed Co. Ltd., Chungbuk, Korea). The quality of obturation was assessed, where only molars with optimum fillings (filling extending till the radiographic apex) or those shorter than the radiographic apex by 1 mm were included and prompted the next step in the trial [20].

In both groups, the access cavity was sealed using a 2-mm layer of self-cured glass ionomer, (GIC; Riva Self Cure, Australia) and finally restored with a bulk-fill composite resin (Tetric N-Ceram Bulk Fill, Ivoclar Vivadent AG, Schaan, Liechtenstein).”

### Assessment of pain severity using Wong-Baker FACES (WBF) pain scale

**Fig. 2:**
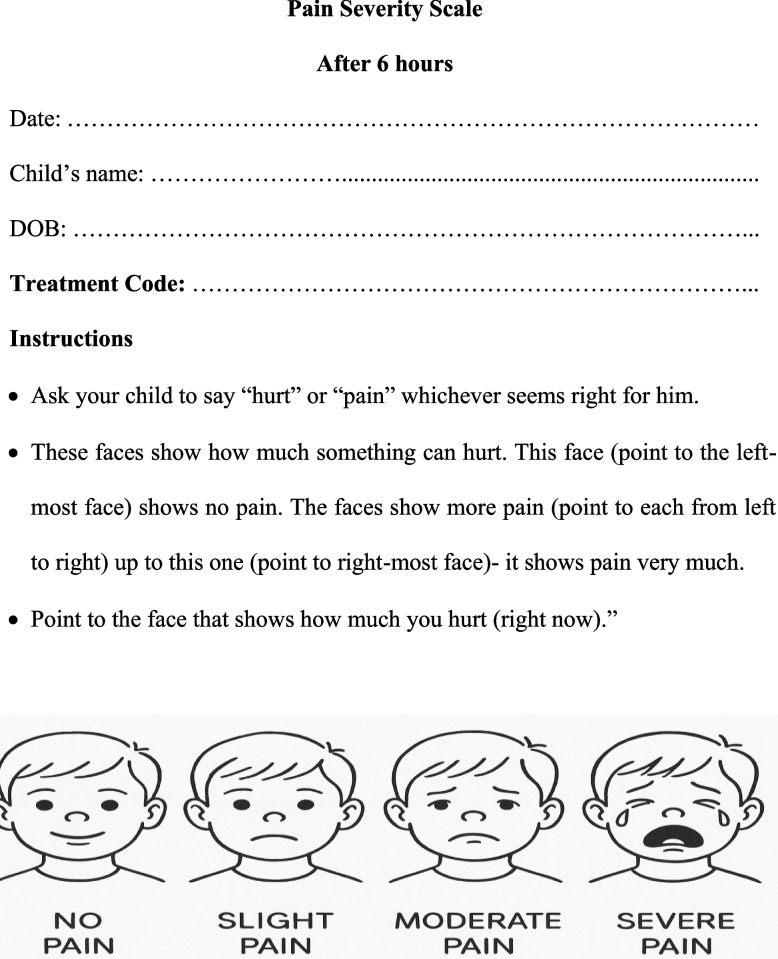
Modified 4-point Wong-Baker Faces pain severity chart

The postoperative severity of pain was determined at 6, 12, 24, 48, 72 h and after one-week following treatment. As shown in Fig. 2, modified Wong-Baker FACES (WBF) pain scale (A 4-point pain intensity scale) [21]. The parents and children received pain-scale training from two independent pediatric dentist specialists, each with fifteen years of experience who were masked to the allocation groups. Six preprinted sheets including the child’s name, date, and treatment code. The instructions were given verbally thoroughly explained to the children’s caregivers and written in the children’s mother tongue as illustrated in Appendix 1. One week later, a statistician who was blind to the research groups examined the data received the data that had been gathered by an independent nurse who was also blind to the experiment. Modified WBF shows four faces of pain-scale assessment with related scores ordered hierarchy from left to right as follows: no pain (score 1), slight pain (score 2), moderate pain (score 3), and severe pain (score 4) [3, 22].

Analgesics were prescribed for each child. Parents were advised not to give painkillers to their children except in cases of absolute necessity. Participants were provided with a standardized analgesic regimen for ethical and methodological reasons. Based on baseline weight, caregivers received ibuprofen (7.5 mg/kg per dose, every 8 h as needed) as the study analgesic. Caregivers received a dosing chart, instructions to record every dose and pain score in a diary. Analgesic intake frequencies and timing was recorded and included as a covariate in adjusted analyses.

### Statistical data analysis

Data analyses were performed using the SPSS statistics software (version 20; IBM SPSS Inc.) and MedCalc – Easy-to-use statistical software https://www.medcalc.org/. The frequencies of participant’s sex, pain intensity score items, and analgesic intake were statistically compared using the chi-square and Fisher’s Exact tests. Continuous data were analyzed using independent sample *t-test*. The outcomes of pain intensity scores were dichotomized into two outcomes in case of pain absence for “no pain scores” and pain presence for “slight pain” scores, “moderate pain” scores, and “severe pain” scores. Considering an equivalence margin of 10% between the two endodontic techniques, the absolute risk difference (ARD) was calculated at a 90% confidence interval. A 90% CI was used because equivalence testing relies on two one-sided tests (TOST) at the 5% level. At less than 5%, the level of significance was adjusted.

## Results

Of one hundred twenty-two children examined for eligibility, 96 children who have met the inclusion standards have been selected. However, four children’s parents refused to participate in the trial. The remaining 92 children who accepted to continue in the study were randomly allocated into two balanced groups (46/group). Regarding the demographic characteristics, no significant sex predilection between the two groups (*p* = 0.834). The mean ages of the pulpotomy and pulpectomy groups were 5.54 ± 0.69 years and 5.59 ± 0.72 years with no significant difference between the two groups (*p* = 0.768) (Table 1).

**Table 1:** Demographic variables of the participants treated with pulpotomy (46 teeth) and pulpectomy (46 teeth)

Variables	Pulpotomy N (%)	Pulpectomy N (%)	*p*-value*
Sex			
Boys	24 (52.2)	25 (54.3)	0.834
Girls	22 (47.8)	21 (45.7)	
Age (years)			
﻿Mean ± SD	5.54 ± 0.69	5.59 ± 0.72	0.768

^*^Chi-square test; *p* < 0.05

**Fig. 3:**
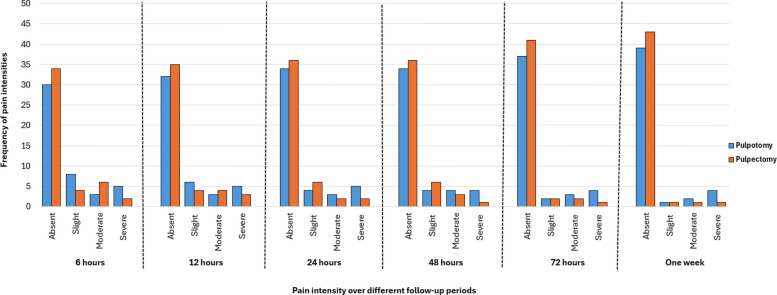
Frequency of pain intensity scores across the follow-up periods

Pain intensities over the follow-up intervals were comparable with no significant differences between the two endodontic techniques. Data in Fig. 3 showed the distribution of pain intensities over the follow-up periods. After six hours, 65.2% of children treated with pulpotomy and 73.9% of children treated with pulpectomy reported no pain, while severe pain was more frequent in the pulpotomy group (10% vs. 4.3%), with no significant difference (*p* = 0.276). The incidence of pain absence after 24 and 48 h was similar (73.9% in the pulpotomy versus 78.3% in the two groups. Pain was absent in 73.9% and 78.3% of children were treated with pulpotomy and pulpectomy, respectively after 24 and 48 h. After 48 h and up to one week, four children (8.7%) treated with pulpotomy suffered from severe pain compared to one patient (2.2%) in the pulpectomy group. These patients were rescheduled for pulpectomy retreatment.

Data in Table 2 showed the frequencies of analgesic intake after pulpotomy were higher compared to those treated with pulpectomy. However, the difference was not significant over the whole follow-up intervals.

**Table 2:** Distribution of analgesic intake after pulpotomy and pulpectomy (N = 46 teeth per group)

Antalgics intake	Pulpotomy N (%)	Pulpectomy N (%)	*p*-value*
After 6 h			
Yes	13 (28.3)	8 (17.4)	0.214
No	33 (71.7)	38 (82.6)	
After 12 h			
Yes	13 (28.3)	6 (13.0)	0.071
No	33 (71.7)	40 (87.0)	
After 24 h			
Yes No	11 (23.9) 35 (76.1)	5 (10.9) 41 (89.1)	0.099
After 48 h			
Yes	10 (21.7)	4 (8.7)	0.072
No	36 (78.3)	42 (91.3)	
After 72 h			
Yes	7 (15.2)	3 (6.5)	0.158
No	39 (84.8)	43 (93.5)	
After one week			
Yes	4 (8.7)	1 (2.2)	0.181
No	42 (91.3)	45 (97.8)	

^*^Chi-square test for 6, 12, and 24 h of follow-up periods and Fisher’s Exact test for 48-, 72-, and one-week follow-up periods; *p* < 0.05

The absolute risk difference between the pulp therapy techniques was not exceedingly the predetermined margin of equivalence at any point of the follow-up with no significant difference between the two groups regarding pain intensity after treatment with pulpotomy or pulpectomy. This indicated that pain intensity scores were equivalent after treatment with pulpotomy compared to pulpectomy of molars with SIP (Table 3).

**Table 3:** Absolute risk difference (ARD) between the pain intensity scores after treatment with pulpotomy and pulpectomy

Follow-up period	Pain absence/presence	Pulpotomy	Pulpectomy	ARD	90% *CI* of ARD	*p*-value*
6 h	Absent	30 (65.2)	34 (73.9)	$-$ 0.087	$-$ 0.244;	0.365
	Presence	16 (34.8)	12 (26.1)		0.070	
12 h	Absent	32 (69.6)	35 (76.1)	$-$ 0.065	$-$ 0.217;	0.482
	Present	14 (30.4)	11 (23.9)		0.087	
24 h	Absent Present	34 (73.9) 12 (26.1)	36 (78.3) 10 (21.7)	$-$ 0.044	$-$ 0.190; 0.102	0.625
48 h	Absent	34 (73.9)	36 (78.3)	$-$ 0.044	$-$ 0.190;	0.625
	Present	12 (26.1)	10 (21.7)		0.102	
72 h	Absent	37 (80.4)	41 (89.1)	$-$ 0.087	$-$ 0.209;	0.385
	Present	9 (19.6)	5 (10.9)		0.035	
One week	Absent	39 (84.8)	43 (93.4)	$-$ 0.086	$-$ 0.192;	0.315
	Present	7 (15.2)	3 (6.6)		0.019	

^*^Chi-square test for 6, 12, 24 and 48 h of follow-up periods and Fisher’s Exact test for 72-, and one-week follow-up periods; *p* < 0.05

## Discussion

The null hypothesis *(H*_*0*_*)* of the current equivalent randomized controlled trial was that treatment of the mandibular second primary molars with SIP with pulpotomy and pulpectomy were not equivalent. While the alternative hypothesis suggested that both treatments were equivalent. The outcomes of the study supported the claim that the intensity of postoperative pain after pulpotomy of primary molars with SIP is equivalent to that recorded by children treated with pulpectomy.

Pain relief is the primary goal of treating inflamed pulp tissues. Pulpectomy is the traditional treatment option of primary molars with irreversible pulpitis to siege the spread of infection [23]. However, the complex anatomy of primary root canal system may complicate clinical procedures and prolong the duration of procedures, which necessitate the need for child’s cooperation [24]. Recently, the comparable outcomes of pulpotomy in treatment of permanent teeth with irreversible pulpitis compared to root canal treatment in diminishing the postoperative pain [25]. Because of the comparable outcomes of pulpotomy and pulpectomy of in treatment teeth with irreversible pulpitis, equivalence design was considered in the current trail. Moreover, a recently released systematic review and meta-analysis emphasized the efficacy of pulpotomy as a definitive approach in treating carious permanent teeth with irreversible pulpitis [26]. Accordingly, to benefit from the privileges of pulpotomy in treatment of primary teeth with SIP. These advantages include shorter working time that enhances the child’s tolerance, more conservative, reducing the possible iatrogenic errors, and more cost-effectiveness [27].

The diagnosis of pulp status, especially the state of irreversibility, is a challenging task. Depending on the severity of symptoms as an indicator of the extent of pulpal inflammation (i.e., mild symptoms indicate reversible pulp inflammation, while severe symptoms indicate irreversible pulpitis) is widely accepted [28]. However, the evidence supporting this association is not sufficient. In the current trial, spontaneous pain plus cold TPT This was confirmed by the conclusion of a recently released systematic review [29] that considered spontaneous pain as a valuable parameter for assessment of irreversible pulpitis especially when conjugated with other diagnostic clinical parameters. Additionally, a previous histological analysis tried to correlate the preoperative symptoms severity and pulpal inflammation state [28]. The histological findings showed a high clinical and histological association of 84.4% between clinical and histologic diagnosis.

Standardization was ensured by several steps that included: 1) performing all clinical steps by a single operator to standardize the procedure’s time length, behavior management approaches, and avoid operator-dependent success variability; 5 min; 3) preoperative severity of pain (i.e., only children with moderate or severe pain scores); 4) excluding children with positive dental experiences. Children with unpleasant dental experiences may worsen the pain perceived by those children and overestimate the pain intensity scores; 5) Only children with Frankl’s scores of 3 or 4 were enrolled in the study because their higher pain threshold compared to uncooperative children [30]; and 6) precluding all children with preoperative analgesic medication. Preemptive analgesics such as ibuprofen and acetaminophen plus codeine may increase the pain threshold and subsequently diminish postoperative pain perception.

There is evidence of weak level directly associate the increasing in time before achieving hemostasis and the incidence of postoperative pain [19]. Therefore, in the current study, the mean time to control pulpal bleeding was confined to no more than 5 min. However, this point needs to be thoroughly investigated in future studies concerning chiefly with primary teeth because most of available literature focuses on the permanent teeth.

Pain perception based on subjective self-reporting pain scales is still the corner stone to express the severity of pain and accredited in several previous literatures [3, 4, 31–33]. In the current trial, pain intensity was measured depending on a subjective self-reporting pain scale (i.e., modified WBFs), for the following reasons: 1) its good reliability, content and construct validity, 2) demonstrates high feasibility in clinical settings and possesses sufficient responsiveness to detect changes in pain over time, 3) easy to use for young children from the age of 3 to 18 years [34, 35], Easily interpreted and enables children to express their pain using an ordinal scale, which is more suitable and cognitively manageable than scales requiring a quantitative estimation of pain intensity [4].

No significant difference between the two-pulp therapy approaches over follow-up intervals. The equivalent outcomes of pulpotomy with pulpectomy could be attributed to technique itself. Both pulp therapy techniques have the privilege of minimizing the local tissue pressure and inflammatory mediator levels [25]. Regarding pulpotomy, the antimicrobial action of MTA and its ability to liberate cytokines may contribute in minimizing the postoperative pain [36]. Furthermore, the irrigation with NaOCl may contribute to decreasing the postoperative pain because of its antibacterial effect [37]. The findings of Ballal et al. [38] showed significant reduction in postoperative pain scores of pulpotomized permanent molars with asymptomatic irreversible pulpitis after disinfection with 2.5% NaOCl compared to physiological saline.

The motivation behind conducting the current study in children was the comparable findings of postoperative pain intensities of less invasive procedures such as partial pulpotomy and pulpotomy compared to pulpectomy in permanent teeth. For instance, Eren et al. [39] pointed out that there were no significant differences between partial pulpotomy, pulpotomy, and pulpectomy in terms of the proportions of pain intensities, thermal and chewing sensitivity, and the need for postoperative analgesics after treating sixty-six permanent molars in both jaws suffering from irreversible pulpitis. Similarly, a multicenter randomized controlled trial results confirmed that the mean pain intensity values in both groups showed a closely similar trend, remaining comparable over the three-day period, after which the pain had completely resolved in both groups [40].

Up to the available data, very little is known about the severity of pain following pulpotomy of primary teeth with irreversible pulpitis. Meanwhile, previous literature focused on clinical and radiographic outcomes of pulpotomy of primary or permanent molars with irreversible pulpitis. Postoperative pain was incorporated as a part of clinical assessment in terms of its presence or absence. Hence, highlighting some of these studies would be beneficial. For instance, after one week, pain was absent in 100% (20 primary molars) of pulpotomized primary molars capped with MTA [41].

Regarding postoperative pain intensity after pulpectomy procedures, the outcomes of the present trial were in line with previous clinical trial that compared two different rotary kinetics in instrumentation of root canal of primary molars [3]. The incidences of pain absence after 6, 12, 24, 48, 72, one week were 78%, 78%, 80.5%, 81.7%, 90, and 100%. Similarly, another clinical trial, supported the reported the incidences of pain severities of 78.1% after 6 and 12 h, 83.6% after 24 h, 93.2% after 48 h, 98.6% after 72 h, which agreed with the current study findings. However, it was necessary to highlight that these two studies assessed the postoperative pain intensity after pulpectomy of necrotic primary molars with no periapical pathosis.

The main strengths of the current prospective clinical trial were 1) the enough participants to declare the differences between the two pulp therapy techniques and 2) Considering restrict standardization measures ensured higher internal validity, prompted replicability, and provided homogenous data that enhancing data analysis. However, highly standardized measures might influence the ability to generalize the findings. For instance, the tooth type and location may have an impact on the severity of postoperative pain perception. Meanwhile, the findings of a previous study revealed that tooth type and location had no impact of the intensity of postoperative pain perception [4]. Another limitation could be related to the restricted age range. Although developmental differences might influence how children perceive and report pain, the evidence is inconsistent. Some studies show higher, more reliable self-reports in older children while others find no independent age effect once preoperative pain and treatment factors are considered [42]. However, considering children aged 5 to 7 years in the present trial was to ensure that children were developmentally capable of self-reporting pain reliably.

Finally, more prospective randomized clinical trials are required to prompt evidence of using pulpotomy as an alternative to pulpectomy in treating primary teeth with SIP, considering a wider age range and taking into account the potential impact of cultural and ethnic dimensions of pain perceived by children. Considering histological studies on pulp tissues of primary teeth are required for better understanding and determination the preoperative pulp status, which has a significant impact on the outcomes of pulp therapy in primary teeth.

## Conclusions

Within the limitations of the current clinical trial, it can be concluded that:

1. Pulpotomy might be an alternative approach to treat primary molars with SIP when hemostasis can be achieved.
2. The incidence of postoperative pain severity after pulpotomy of the mandibular primary molars with SIP were equivalent to those treated with pulpectomy over the follow-up periods.
3. The need for postoperative analgesics was comparable after treatment of primary molars with SIP either with pulpotomy or pulpectomy.

## Supplementary Information

Supplementary Material 1.

Supplementary Material 2.

## Data Availability

All data generated or analyzed during this study are included in the article.

## Abbreviations

VPT: Vital pulp therapy
SIP: Symptomatic irreversible pulpitis
CONSORT: Consolidated Standards of Reporting Trails
ARD: Absolute risk difference
IANB: Inferior alveolar nerve block
MTA: Mineral trioxide aggregate
WBF: Wong-Baker FACES
NaOCl: Sodium hypochlorite
