# Supplementary material for: Postoperative pain after pulpotomy versus pulpectomy of primary molars with symptomatic irreversible pulpitis: an equivalent randomized clinical trial
Source: BMC Oral Health. 2026 Apr 25;26:786. doi: 10.1186/s12903-026-08329-z (PMC13141260; doi:10.1186/s12903-026-08329-z)
Supplement: Supplementary file 1 — Supplementary Material 1. [file 12903_2026_8329_MOESM1_ESM.pdf]

Appendix 1

Pain severity scale (after 6 hours)
مقياس شدة الألم بعد 6 ساعات

Date: ………………………………
Child's name: ……………………...
DOB: ………………………………

التاريخ: ....
اسم الطفل: ....
تاريخ الميلاد: ....

Treatment code: A

Instructions

- Ask your child to say "hurt" or "pain" whichever seems right for him.
- These faces show how much something can hurt. This face (point to the left-most face) shows no pain. The faces show more pain (point to each from left to right) up to this one (point to right-most face)- it shows pain very much.
- Point to the face that shows how much you hurt (right now)."

تعليمات

- اطلب من طفلك أن يقول "ألم" أو "يوجعني" حسب الكلمة التي تناسبه
- هذه الوجوه تُظهر مدى شدة الألم الذي يمكن أن يشعر به الإنسان. هذا الوجه (أشر إلى الوجه الموجود في أقصى اليسار) يُظهر عدم وجود ألم. وكلما اتجهنا نحو اليمين، تزداد شدة الألم تدريجيًا (أشر إلى كل وجه من اليسار إلى اليمين) حتى نصل إلى هذا الوجه (أشر إلى الوجه الموجود في أقصى اليمين) – وهو يُظهر ألمًا شديدًا جدًا.
- أشر إلى الوجه الذي يُعبّر عن مقدار الألم الذي تشعر به الآن

For girls خاص بالأطفال الاناث (البنات)

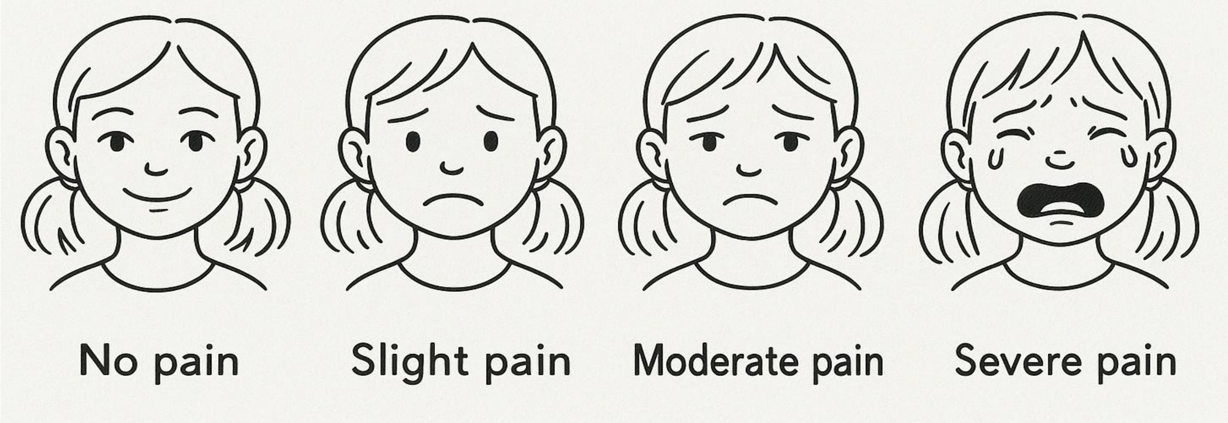

لا أشعر بأي ألم | **ألم خفيف** | **ألم متوسط (محتمل)** | **ألم شديد (غير محتمل)**

Modified 4-point Wong-Baker Faces pain severity charts for girls
